# On the Non-Contagiousness of Cancer

**Published:** 1890-12-06

**Authors:** 


### ON THE NON-CONTAGIOUSNESS OF CANCER.

Dr. Walter K. Sibley read a paper on the Noncontagiousness of Cancer. The words "infection" and "contagion" were first defined, as they were used in his communication. He mentioned that an element, namely "germs," was common to both; in infection the germs were capable of transmission by floating in the air or through the medium of fomites; but in contagion they were inseparable from the animal products in which they were thrown off from the diseased part; that is to say, they were only capable of transmission by direct contact. The classification was artificial, but the distinction was convenient. Some supposed cases of contagion of cancer were next described. When two surfaces were in contact, a primary cancer of the one was sometimes followed by a secondary cancer of the other, such as in the lips, vocal cords, the labia of the female genital organs, the opposite surfaces of the urinary bladder, &c. Some observers had even believed in the spread by contagion from one person to another, and quoted in support some cases of malignant diseases. The author proceeded to say, it was well known that the seat of a primary cancer was often determined by chronic irritation, as on the lips, scrotum, intestine, gall bladder, and urinary bladder. The occurrence of a secondary growth opposite a primary one, must be due, either to direct contagion, or to constant irritation. But as the seat of a primary growth was no doubt determined by local irritation, still more might it be expected that the seat of a secondary deposit should be determined by the same cause. For instance, the presence of an epitheliomatous ulcer on one lip might itself be sufficient mechanical irritation to the region of the other lip, in constant contact with it, to cause the development of a secondary growth there; not through any special or specific contagious property of the carcinomatous growth itself, but simply because of the irritation of the opposite lip produced by its presence. Dr. Sibley next described the case of a man with an epitheliomatous ulcer around the base of a carious tooth, and who had a small ulcer of the same nature on the inside of the cheek. This second ulcer did not come in contact with the primary one, but rested against the crown of the same tooth. It appeared that the seat of the primary ulcer had been determined by the tooth, and of the second one by the tooth also; if the two ulcers had been in contact themselves, it might have appeared to be a case of inoculation of the primary to the secondary cancerous growth. He then referred to the inoculation experiments of cancer into lower animals, the results of all of which were very doubtful, and noticed especially those of Messrs. Ballance and Shattock.* The experiments of these last observers were of the character of transplantations rather than of inoculations, and as the result of them the authors stated that "all the experiments had yielded negative results." Concerning the well-known experiments of Lagenbeck, who is stated to have produced cancer in a dog from the inoculation of cancer juice from a case of cancer in a man, he quoted Virchow† who says "I have myself examined the microscopical specimens of the case of lung tumour. They have more the appearance of that form of spontaneous cancer I have myself found in the dog than that of the cancer elements as found in man." After referring to the so-called cancer bacillus of Scheuerlen of Berlin, which had turned out to be the ordinary potato bacillus, and to the statements of Kubasoff, of Moscow, the author summed up his paper by saying, "he believed that secondary growths of cancer sometimes occurred started by primary ones, not through any contagious properties of the latter, but merely from the mechanical irritation these cause," and then quoted Mr. Jonathan Hutchinson ‡ to the effect that cancer was not due to the introduction of any special material from without into the system: there was nothing specific in its nature; it was simply a modification of what occurred in chronic inflammation. Cancer occurred in old people in an organ which had become old; for instance, the mammary gland in the female grows old before the rest of the body. In senility chronic inflammation with overgrowth of tissue was very common, and as a variety of this cancer also frequently occurred. It sometimes happened that in very old people all the stages between chronic inflammation and cancer were seen in the same individual, so that it was not always easy to say which processes were inflammatory and which cancerous.

Dr. Dickinson remarked that there was published in the "Transactions of the Society" for 1861 the case of a woman who had a malignant cyst of the ovaries. This tumour had burst into the abdominal cavity, and a quantity of pieces of cancer had apparently become adherent to the walls of the intestines. The older ones were firmly attached, and the recent only just adherent, and from this case it would appear that it is possible for pieces of cancer to become inoculated in the same individual.

Mr. Hutchinson referred to the case of a man with an epithelioma of the lower lip, and who had on the back of the hand a growth of a similar nature. Another case he remembered was one in which there were several isolated tumours for some inches of the intestine, as if they had spread from one to the other.

Mr. Roger Williams failed to see the connection between these cases and the point in question, and said there was no form of tumour that might not have more than one initial spot of development. He nevertheless believed in auto-inoculation.

Mr. Shattock showed a photograph of the case of supposed inoculation of cancer into a rat by Hanau, and stated that the only cases of successful inoculation had been those of cancer of a similar nature and in the same kind of animals. Cancer was sometimes strictly local in origin, but it might be a general disease. Mr. Spencer referred to the cases of Hanau's rats, and said that many of them were affected with cancer before they were inoculated at all, either from all being kept together, or at any rate there must have been a very strong tendency to the development of cancer in these particular rats quite apart from any process of inoculation.

Dr. Walter Sibley, in reply, said he did not believe in the possibility of inoculation, or in auto-inoculation of cancer; but that when a secondary cancer formed in contact with a primary one, it was the result of the irritation of the tumour, and not from any contagiousness of the same.

* Proceedings of the Royal Society, vol. 48.

† Virchow's Krankhafte Geschwulste, Bd. 1, s. 87.

‡ The Hospital, Vol. 9, p. 43.

